# Interventions for Children Affected by Armed Conflict: a Systematic Review of Mental Health and Psychosocial Support in Low- and Middle-Income Countries

**DOI:** 10.1007/s11920-015-0648-z

**Published:** 2016-01-14

**Authors:** Mark J. D. Jordans, Hugo Pigott, Wietse A. Tol

**Affiliations:** Center for Global Mental Health, Institute of Psychology, Psychiatry & Neuroscience, King’s College London, London, UK; Research and Development Department, War Child Holland, Amsterdam, the Netherlands; Center for Global Mental Health, London School of Hygiene and Tropical Medicine, London, UK; Department of Mental Health, Johns Hopkins Bloomberg School of Public Health, Baltimore, USA

**Keywords:** Armed conflict, Children, Evidence base, Systematic review, Violence, War

## Abstract

Over one billion children under the age of 18 live in countries affected by armed conflict. This systematic review replicates an earlier study, aiming to provide a comprehensive update of the most current developments in interventions for children affected by armed conflict. For the period 2009–2015, a total of 1538 records were collected from PubMed, PsycINFO, and PILOTS. Twenty-four studies met the inclusion criteria, and the included interventions involve data from 4858 children. Although the number of publications and level of evidence has improved since the previous review, there is still a general lack of rigor and clarity in study design and reported results. Overall, interventions appeared to show promising results demonstrating mostly moderate effect sizes on mental health and psychosocial well-being. However, these positive intervention benefits are often limited to specific subgroups. There is a need for increased diversification in research focus, with more attention to interventions that focus at strengthening community and family support, and to young children, and improvements in targeting and conceptualizing of interventions.

## Introduction

Over one billion children under the age of 18 live in countries affected by armed conflict [1]. In 2013, 33 armed conflicts were recorded, with the majority in Africa (39 %), Asia (39 %), and the Middle East (12 %) [2]. These conflicts take place principally in “lower and middle-income countries” (LMIC), where 90 % of the world’s population of children and adolescents live.

Mental health and psychosocial well-being in settings of armed conflict are threatened by exposure to violence and other risk factors for mental health such as increased poverty and lack of access to basic services for example healthcare, education, housing, water, and sanitation [3]. In an armed conflict situation, the population is affected in various ways and consequently requires different kinds of support. International consensus guidelines, such as the Inter-Agency Standing Committee (IASC) guidelines for mental health and psychosocial support (MHPSS) in emergencies, agree on the need for a multi-layered system of support that is delivered at different levels of social and health systems [4]. The framework emphasizes integrating different forms of support, ranging from activities for the population as a whole (for example, providing general humanitarian support with respect to dignity and in a participatory manner), non-specialized activities that strengthen protective factors (for example, with a focus on strengthening informal social supports and existing coping mechanisms), and programs that address a smaller percentage of the population displaying significant psychological distress or mental disorders, for whom more specialized support is needed [5]. In public health terms, these interventions can be divided into promotion (i.e., activities aimed at strengthening positive aspects of mental health and well-being), prevention (i.e., activities aimed at making sure mental health problems do not develop, for example through action on the social determinants of mental health), and treatment (i.e., activities to reduce symptoms and improve functioning in people with identified mental disorders) [6].

Despite an increase in efforts to evaluate MHPSS for children and adolescents in areas of armed conflict, there remain important gaps in evidence [7]. A previous systematic review on psychosocial and mental health interventions for children in areas of armed conflict was published in 2009. That review concluded that there is a paucity of rigorous studies. Existing studies focused predominantly on PTSD as an outcome of interventions, and outcomes of evaluations were mixed. Given the time passed since this review, an update is timely [8]. Such an update may be useful in determining where the current focus of interventions lies and whether there are changes in types of interventions that are commonly implemented, as well as their effectiveness. For example, several authors have called in the past for paradigm shifts in MHPSS interventions for populations affected by armed conflict [9, 10]. Existing consensus guidelines broadly endorse proposed shifts away from a single focus on treatment of psychological symptoms to contextually appropriate multi-layered systems of support that build on existing resources. However, we previously did not find such broadening of interest reflected in the published peer-reviewed literature.

The purpose of this current systematic review is to assess the evidence of interventions since 2009, providing a current state of the art overview. The objective is to examine the type and effectiveness of psychosocial and mental health interventions for conflict-affected children. All study designs were assessed in order to broadly summarize the evidence for MHPSS.

## Method

### Study Selection

The inclusion and exclusion criteria for studies are listed in detail in Appendix 1. In summary, the review includes all studies that describe, and evaluate the effect of, psychosocial and mental health interventions for children affected by armed conflict in LMIC. The composite term MHPSS is used to describe any type of local or outside support that aims to protect or promote psychosocial well-being and/or prevent or treat mental disorder [4].

### Data Sources and Searches

The search was restricted by language, publication status, and date. The following electronic databases were used: PubMed/MEDLINE, PsycINFO, and PILOTS from January 1, 2009 to July 20, 2015. The following search terms were used: (child* OR adolescent*) AND (war* OR “community violence” OR “armed conflict”) AND (mental health OR psychosocial) AND (intervention OR treatment). In addition, bibliographies of eligible papers were manually examined for relevant citations our searches missed. The authors of included studies were contacted in the event of missing data. After conducting the searches, we first screened all titles and abstracts for meeting inclusion and exclusion criteria outlined above (by HP and cross-checked by MJ). The remaining papers were fully read by two authors to check for papers including MHPSS interventions that assessed an outcome for children affected by armed conflict in LMIC.

### Data Extraction and Risk of Bias Assessment

Data from these selected papers was obtained by using a previously developed [8] standard data extraction form. The study characteristics extracted were theoretical framework, specification of target groups and descriptions of interventions, treatment modalities, methodologies, and outcomes. The methodological quality of included studies was assessed by one person using the Cochrane risk of bias tool [11]. Risk of bias was assessed at both study and outcome levels.

### Data Synthesis and Analysis

In accordance with our study aims, we conducted two types of analysis. First, for an account of intervention descriptions, we used thematic analysis to summarize themes, with a specific focus on cultural adaptations. Second, all evaluation studies reporting quantitative data were categorized into level of evidence (1 = randomized controlled trials, all types; 2 = quasi-experimental design and controlled studies; 3 = non-controlled design; 4 = case studies; adapted from Morris) [12]. Also, interventions were categorized according to the different levels of the pyramid of the IASC guidelines for mental health and psychosocial support in emergencies (i.e., social considerations in basic services and security, strengthening community and family supports, focused non-specialized support, specialized services) [4]. To summarize quantitative evaluation studies, Cohen’s *d* effect size calculations were used to obtain an indication of strength of intervention benefits and allow for a comparison of the strength of intervention benefits (or harms) across interventions. Effect sizes were not adjusted for effects of clustering in cluster randomized trials. Effect sizes were graded as less than 0.30 small, 0.30 to 0.60 moderate, and above 0.60 large [13]. The validity of the quantitative evaluation studies was assessed using the Cochrane risk of bias tool [11]. It includes categorical variables 1–7 to quantify selection bias consisting of random sequence generation and allocation concealment (1–2, respectively), performance bias comprising of blinding of participants and personnel (3), detection bias consisting of blinding of outcome assessment (4), attrition bias detailing incomplete outcome data (5), reporting bias comprising of selective reporting (6), and other sources of bias consisting of uncovered problems (7). These categorical variables were scored by low risk, high risk, and unclear risk. The methodology and results are presented according to the PRISMA statement for reporting systematic reviews [14].

## Results

**Fig. 1:**
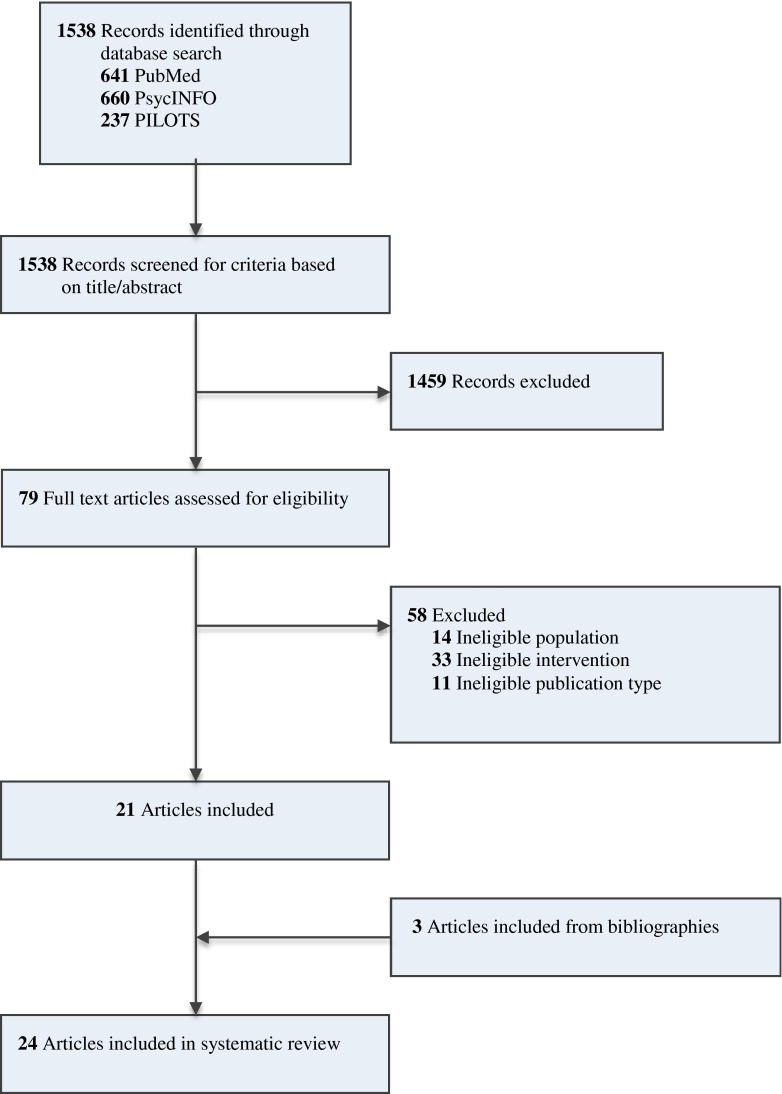
Study flowchart

Figure 1 shows the screening and selection procedure for this review. A total of 1538 records were collected from three databases. Twenty-four studies met the inclusion criteria. Four studies used the same data set [15-18]. Table 1 provides an overview of all included studies.

**Table 1:** Evidence base (*n* = 24, consisting of 20 interventions)

Reference, country	Age (years), gender, sample	Modality (level), focus	Delivery	Level	Bias	Training (T) and supervision (S)	Cultural adaptations	Primary outcome (PO) and components (C)	Results (R) and effect size (*d*)
Ager (2011) [29], Uganda	7–12, 50 % M and 50 % F, *N* = 403	Level 2, well-being	15 group sessions for 5 weeks delivered by non-specialist (teacher)	2	1 = high	T = yes S = not stated	Intervention incorporates needs of child and local community Instruments created through FGD with children, parents, and teachers	PO = well-being C = school-based recreational and connectivity activities	R = well-being improved more in experimental group compared to control. Predictors for well-being were group assignments and age. *d* = 0.18–0.64 (well-being)
					2 = unclear				
					3 = high				
					4 = high				
					5 = high				
					6 = unclear				
					7 = high				
Barron (2012) [35], Palestine	11–14, 59 % M and 41 % F, *N* = 140	Level 3, PTSS/depression/grief	5 group sessions for 5 weeks delivered by non-specialist (counselor)	1	1 = low	T = counselor 3 days S = observed classes	Intervention translated in Arabic Instrument adaptations not stated	PO = PTSS and depression C = school-based TF-CBT	R = significant decreases across all indicators in experimental group compared to control. Students reported their experiences as very positive. *d* = 0.76 (PTSS) *d* = 1.24 (depression) *d* = 0.90–0.96 (emotion and behavior)
					2 = unclear				
					3 = unclear				
					4 = unclear				
					5 = high				
					6 = unclear				
					7 = high				
Betancourt (2012) [36], Uganda	14-17, 43 % M and 57 % F, *N* = 304	Level 3, depression	16 group sessions for 16 weeks delivered by non-specialist (facilitators)	1	1 = low	T = facilitator 2 weeks S = weekly	Intervention adapted and tested and qualitative work shows compatibility with target group Instruments based on previous qualitative work on same sites	PO = moderators of depression C = community-based G-IPT focused on triggers and group relationships	R = gender and abduction moderated the effectiveness of G-IPT on depression. Female non-abducted greatest improvement and male non-abducted had a negative impact. *d* = 1.06 (female non-abduction) *d* = −0.02 (male non-abduction)
					2 = high				
					3 = low				
					4 = low				
					5 = high				
					6 = unclear				
					7 = low				
Claessens (2012) [30], Uganda	10–15, not stated, *N* = 510	Level 2, well-being	Group sessions for 17 weeks delivered by non-specialist (social worker)	3	1 = high	T = social worker 1 day S = not stated	Intervention specifically for conflict-affected areas but adaptations not stated Instruments used not clinical, mixed-methods approach	PO = well-being C = school-based recreational and connectivity exercises	R = 54.3 % children achieved their goal. Themed quiz children got 87 % answers correct. Interaction helped build better relationships and reported improved well-being. *d* = N/A
					2 = high				
					3 = high				
					4 = high				
					5 = high				
					6 = unclear				
					7 = high				
Diab (2014) [17], Palestine	10–13, 51 % M and 49 % F, *N* = 482	Level 3, social support impacting PTSS/depression	Time frame not stated, delivered by non-specialist (counselor)	1	1 = low	T = yes S = weekly	Intervention previously found to be effective in Palestine children but adaptations not stated Instruments used in setting before but adaptations not stated	PO = peer and sibling relations C = school-based TF-CBT	R = gender-specific results. Loneliness in peer relations reduced among boys and sibling rivalry among girls. Intervention decreased mental health problems by improving peer relations. *d* = N/A
					2 = unclear				
					3 = unclear				
					4 = unclear				
					5 = high				
					6 = unclear				
					7 = high				
Diab (2015), [15] Palestine	10–13, 51 % M and 49 % F, *N* = 482	Level 3, social support impacting resiliency	Time frame not stated, delivered by non-specialist (counselor)	1	1 = low	T = yes S = weekly	Intervention found to be effective in Palestine children previously but adaptations not stated Instruments used in setting before but adaptations not stated	PO = resilience and moderating role of family factors C = school-based TF-CBT	R = did not increase resilience (defined as prosocial behavior and well-being) but improved quality of peer and sibling relations. *d* = N/A
					2 = unclear				
					3 = unclear				
					4 = unclear				
					5 = high				
					6 = unclear				
					7 = high				
Eiling (2014), [31] South Sudan	8–16, 60 % M and 40 % F, *N* = 122	Level 2, well-being	19 group sessions for 4–6 months delivered by non-specialist (community worker)	3	1 = high	T = community worker 3 days S = not stated	Intervention specifically for conflict-affected areas but adaptations not stated Instruments used not clinical, mixed-methods approach	PO = well-being C = school-based recreational and connectivity exercises	R = 48 % respondents stated they noticed significant personal improvements. Main changes reported were decreased fighting and improved relationships. *d* = N/A
					2 = high				
					3 = high				
					4 = high				
					5 = high				
					6 = unclear				
					7 = high				
Hasanovic (2009), [32] Bosnia and Herzegovina	12–15, 32 % M and 68 % F, *N* = 408	Level 3, PTSS	20 group sessions for 20 weeks delivered by non-specialist (student, teacher, parent)	2	1 = high	T = students 15 h, teachers 15 h, parents 14 h S = not stated	Intervention modified to match participants needs Instruments adaptations not stated	PO = PTSS C = school-based psycho-educative and expressive classes	R = PTSS decreased significantly in experimental group compared to control. Indication PTSS may be transmitted through caregivers. *d* = 0.14 (PTSS)
					2 = high				
					3 = high				
					4 = unclear				
					5 = low				
					6 = unclear				
					7 = high				
Jordans (2010), [24] Nepal	11–14, 51 % M and 49 % F, *N* = 325	Level 3, PTSS/depression/behavioral and emotional problems	15 group sessions for 5 weeks delivered by non-specialist (interventionists)	1	1 = low	T = interventionists 15 days, local researcher 3 weeks S = regularly	Intervention adaptations not stated Instruments translated and tested for reliability with subgroup	PO = PTSS and depression C = school-based creative expressive focused CBT with distinct trauma focus	R = no main effects found, but several moderate subgroup effects on prosocial behavior, sense of hope, aggression, and psychological difficulties. The intervention improved generic psychosocial problems. *d* = 0.1 (PTSS) *d* = 0.46 (depression)
					2 = unclear				
					3 = high				
					4 = high				
					5 = low				
					6 = unclear				
					7 = unclear				
Jordans (2011), [19] Burundi/Indonesia/Sri Lanka/Sudan	7–15, N/A, N/A	Level 2/3, N/A	Different durations delivered by non-specialist (community workers, facilitators, counselor)	4	1 = N/A	T = community worker 1/2 weeks, facilitator 3 weeks, counselor 4 months S = not stated	N/A	PO = N/A C = presents a multi-layered model for community-based care following public health approach	R = multi-layered system is feasible, high levels of client satisfaction; post-treatment positive changes reported. Relatively high distress to service providers *d* = N/A
					2 = N/A				
					3 = N/A				
					4 = N/A				
					5 = N/A				
					6 = N/A				
					7 = N/A				
Jordans (2012), [21] Burundi	11–14, 18 % M and 82 % F, *N* = 11	Level 3, mechanisms of counseling	8 individual sessions for 8 weeks delivered by non-specialist (counselor)	4	1 = high	T = counselor 3 months, local researcher 4 weeks S = not stated	Intervention adaptations not stated Instruments translated and scales created through qualitative work	PO = treatment mechanisms C = community-based counseling focus on individual empowerment	R = Positive results associated with counselor demonstrating reflective involvement, absence moralistic behavior, opportunity to express emotions and inclusion of family. *d* = N/A
					2 = high				
					3 = high				
					4 = high				
					5 = low				
					6 = unclear				
					7 = high				
Jordans (2013), [25] Burundi	10–14, 49 % M and 51 % F, *N* = 97	Level 3, depression/behavioral problems/social support	2 group sessions delivered to parents by non-specialist (counselor)	2	1 = high	T = counselor 3 months, local assessor 2 days S = not stated	Intervention adaptations not stated Instruments translated, back translated and FGDs used	PO = depression and aggression C = community-based mental health problem management strategies focus psycho-education	R = reduced aggression compared to control, especially among boys. Did not show impact on depression symptoms and parents evaluated the intervention positively. *d* = 0.08 (depression) *d* = 0.60 (aggression)
					2 = unclear				
					3 = unclear				
					4 = unclear				
					5 = low				
					6 = unclear				
					7 = high				
Jordans (2013), [20] South Sudan	10–15, 33 % M and 67 % F, *N* = 6	Level 3, mechanisms of counseling	8 individual sessions for 8 weeks delivered by non-specialist (counselor)	4	1 = high	T = counselor 3 months, local researcher 4 weeks S = not stated	Intervention adaptations not stated Instruments translated and scales created through qualitative work	PO = treatment mechanisms C = community-based counseling focus on individual empowerment	R = positive results associated with quality of relationship, level of client activation, and ability of counselor to match treatment to problems. *d* = N/A
					2 = high				
					3 = high				
					4 = high				
					5 = low				
					6 = unclear				
					7 = high				
McMulle (2013), [22] Democratic Republic of the Congo	13–17, 100 % M, *N* = 50	Level 3, PTSS/internalizing symptoms/behavioral problems	15 group and one-to-one sessions delivered by specialist and non-specialist (counselor, author)	1	1 = low	T = daily S = evaluation sessions held daily	Intervention adapted and modified including culturally applicable analogies and exemplars throughout Instruments translated and FGDs used	PO = PTSS, depression, and anxiety C = community-based TF-CBT incorporating coping and processing skills	R = highly significant decreases across all indicators in experimental group compared to control. Treatment gains maintained at 3-month follow-up. *d* = 2.75 (PTSS) *d* = 2.13 (depression/anxiety) *d* = 1.28–2.46 (emotion and behavior)
					2 = low				
					3 = unclear				
					4 = unclear				
					5 = low				
					6 = unclear				
					7 = unclear				
O’Callaghan (2013), [23] Democratic Republic of the Congo	12–17, 100 % F, *N* = 52	Level 3, PTSS/internalizing symptoms/behavioral problems	15 group and one-to-one sessions by non-specialist (social worker, evaluator)	1	1 = low	T = yes S = evaluation sessions held daily	Intervention adapted and modified through daily meetings with community involvement Instruments translated, FGDs used and piloted	PO = PTSS, depression, and anxiety C = community-based TF-CBT incorporating coping and processing skills	R = highly significant decreases across all indicators in experimental group compared to control. Continual improvements after 3 months. *d* = 2.01 (PTSS) *d* = 2.01 (depression/anxiety) *d* = 0.64–1.10 (emotion and behavior)
					2 = low				
					3 = unclear				
					4 = low				
					5 = low				
					6 = unclear				
					7 = unclear				
O’Callaghan (2014), [33] Democratic Republic of the Congo	7–18, 55 % M and 45 % F, *N* = 159	Level 2, PTSS/internalizing symptoms/behavioral problems	8 group sessions for 3 weeks, delivered by non-specialist (local lay workers)	1	1 = low	T = local lay worker 2 days S = evaluation sessions held daily	Intervention adapted with community leaders and facilitators to assess impact and make cultural changes Instruments translated, back translated and piloted	PO = PTSS, depression, and anxiety C = community-based psycho-educative classes focus on communication and resolution	R = Moderate reductions across all indicators in experimental group compared to control. Similar results 3 month follow-up. *d* = 0.40 (PTSS) *d* = 0.09 (depression/anxiety) *d* = 0.08–0.22 (emotion and behavior)
					2 = low				
					3 = low				
					4 = low				
					5 = low				
					6 = unclear				
					7 = unclear				
Peltonen (2012), [37] Palestine	10–14, 64 % M and 36 % F, *N* = 225	Level 2, PTSS/internalizing symptoms/behavioral problems	Individual sessions for 8 months delivered by non-specialist (students)	1	1 = low	T = yes S = monthly workshops and sessions	Intervention shown to be effective in Palestine Instruments shown to be reliable with Palestine children Adaptations not stated for intervention or instruments	PO = PTSS, depression, and distress C = school-based psycho-educative focus on communication and resolution	R = small reductions across indicators in experimental group compared to control. However, increase in PTSS in experimental group but not control. *d* = 0.12 (PTSS) *d* = 0.32 (depression) *d* = 0.01–044 (emotion and behavior)
					2 = unclear				
					3 = unclear				
					4 = unclear				
					5 = high				
					6 = unclear				
					7 = high				
Punamaki (2014), [17] Palestine	10–13, 51 % M and 49 % F, *N* = 482	Level 3, emotion regulation impacting mental health	8 group sessions for 4 weeks delivered by non-specialist (counselor)	1	1 = low	T = yes S = weekly meetings	Intervention found to be effective in Palestine children Instruments used in setting before Adaptations not stated for intervention or instruments	PO = emotion regulation C = school-based TF-CBT	R = not effective in changing emotion regulation (ER), and ER did not mediate the intervention effects on children’s MH. A decrease in ER was associated with better mental health. *d* = N/A
					2 = unclear				
					3 = unclear				
					4 = unclear				
					5 = high				
					6 = unclear				
					7 = high				
Qouta (2012), [18] Palestine	10–13, 51 % M and 49 % F, *N* = 482	Level 3, PTSS/depression/distress	8 group sessions for 4 weeks delivered by non-specialist (counselor)	1	1 = low	T = yes S = weekly meeting with author	Intervention effective in children traumatized in war Instruments used in setting before Adaptations not stated for intervention or instruments	PO = PTSS, peri-traumatic dissociation, and depression C = school-based TF-CBT	R = subgroup effects with reduction PTSS among boys, girls had reductions in PTSS if they showed low peri-traumatic dissociation. *d* = 0.13 (PTSS) *d* = −0.09 (depression)
					2 = unclear				
					3 = unclear				
					4 = unclear				
					5 = high				
					6 = unclear				
					7 = high				
Staples (2011), [38] Palestine	8–18, 63 % M and 37 % F, *N* = 129	Level 4, PTSS/depression	10 group sessions for 5 weeks delivered by specialist (mental health professional)	3	1 = high	T = not stated S = not stated	Intervention adaptations not stated but proven to be effective in Kosovo Instruments translated and back translated	PO = PTSS and depression C = community-based traumatic grief psychotherapy	R = significantly reduced PTSS and depression and decreased sense of hopelessness. With gains maintained at 7 months follow-up. *d* = 2.38 (PTSS) *d* = 0.74 (depression) *d* = 0.62 (hopelessness)
					2 = high				
					3 = high				
					4 = unclear				
					5 = high				
					6 = unclear				
					7 = high				
Thabet (2009), [34] Palestine	6–16, 87 % M and 13 % F, *N* = 304	Level 2, behavioral problems	Group sessions delivered by non-specialist (student)	3	1 = low	T = yes S = not stated	Intervention translated into Arabic Instruments developed and validated in Palestine Adaptations not stated for instruments	PO = behavioral problems C = school-based psycho-educative focus on communication and resolution	R = small reductions in behavior and depression, decreases in hyper-activity reported by children. Parents reported decrease in obsessive and over anxious symptoms. *d* = 0.06–0.17 (behavior) *d* = 0.03–0.11 (depression)
					2 = unclear				
					3 = high				
					4 = high				
					5 = unclear				
					6 = unclear				
					7 = high				
Tol (2010), [26] Indonesia	7–15, 51 % M and 49 % F, *N* = 403	Level 3, social support mediating and moderating PTSS	15 group sessions for 5 weeks delivered by non-specialist (counselor)	1	1 = low	T = counselor 3 weeks S = yes	Intervention adaptations not stated Instruments translation and statistical techniques used	PO = hope and social support C = school-based creative expressive techniques combining CBT	R = treatment showed maintained hope and increased social support. Play social support associated with smaller reductions in PTSS. Girls showed larger treatment benefits in PTSS. *d* = N/A
					2 = high				
					3 = high				
					4 = high				
					5 = unclear				
					6 = unclear				
					7 = unclear				
Tol (2012), [28] Sri Lanka	9–12, 61 % M and 39 % F, *N* = 399	Level 3, PTSS/depression/behavioral and emotional problems	15 group sessions for 5 weeks by non-specialist (counselor)	1	1 = low	T = yes S = yes Both for 1 year prior to study	Intervention adaptations not stated Instruments translated, FGDs, and interviews used	PO = PTSS and depression C = school-based creative expressive techniques combining CBT	R = main effect on conduct problems. Several subgroup benefits identified. Negative results PTSS for girls *d* = −0.05 (PTSS) *d* = 0.10 (depression)
					2 = unclear				
					3 = unclear				
					4 = unclear				
					5 = unclear				
					6 = unclear				
					7 = unclear				
Tol (2014), [27] Burundi	8–17, 52 % M and 48 % F, *N* = 329	Level 3, PTSS/depression/behavioral and emotional problems	15 group sessions for 5 weeks by non-specialist (counselor)	1	1 = low	T = yes S = yes Both for 1 year prior to study	Interventions adaptations not stated Instruments translated, FGDs, and interviews used	PO = PTSS and depression C = school-based creative expressive techniques combining CBT	R = no main effects. Six favorable and two unfavorable subgroup effects identified, moderated by age, household composition, exposure, and displacement *d* = −0.02 (PTSS) *d* = −0.03 (depression)
					2 = unclear				
					3 = unclear				
					4 = unclear				
					5 = high				
					6 = unclear				
					7 = unclear				

Modality and level were categorized according to the Inter-Agency Standing Committee mental health and psychosocial support pyramid (level 1 = social considerations in basic health services and security, level 2 = strengthening community and family supports, level 3 = focused non-specialized care, level 4 = specialized services) [19]. Level is the evidence level of study design [12]

*M* male, *F* female, *N* sample size, *PTSS* post-traumatic stress symptoms, *FGDs* focused group discussions, *TF-CBT* trauma-focused cognitive behavioral therapy, *G-IPT* group interpersonal therapy

### Delivery and MHPSS Approach^1^

The collection of publications focuses on armed conflicts in nine countries, with 46 % (*n* = 11) of the studies taking place in Asia, 46 % (*n* = 11) in Africa, 4 % (*n* = 1) in Europe, and 4 % (*n* = 1) in a collection of countries. The included 24 publications identified in peer-reviewed journals had the following study designs: five individually randomized controlled trials (RCTs), nine cluster randomized controlled studies, three controlled studies, four non-controlled studies, and three case studies. One case study summarized results of a collection of studies and reported high levels of client satisfaction, moderate post-treatment problem reductions, and significant levels of distress for service providers [19]. It was included in the analysis but excluded from calculating percentages below.

The included quantitative evaluation studies interventions involved data being collected with 4858 children, with just under two thirds (60 %, *n* = 12) using a school as a delivery platform and over a third (40 %, *n* = 8) implemented in community settings. The interventions consisted of 30 % (*n* = 6) MHPSS level 2 activities, 65 % (*n* = 13) level 3, and 5 % (*n* = 1) level 4 initiatives. A non-specialist (a service provider who did not receive years of training is specialized care) delivered 90 % of these interventions. The duration varied with 55 % (*n* = 11) implemented in 15 sessions or more, 5 % (*n* = 1) had ten to 14 sessions, 30 % (*n* = 6) less than ten sessions, and 10 % (*n* = 2) did not record the number of sessions. Training of a delivery agent was stated in 95 % (*n* = 19) of interventions. Eight programs included less than 1 month’s training for the delivery agent; however, these were for individuals with extensive prior experience working with the study population. Three interventions included 3 months of training. Of all programs, 55 % (*n* = 11) included supervision in order to support those implementing interventions and ensure fidelity to the program. There were seven interventions (35 %) that implemented MHPSS regardless of children’s symptoms (universally), and 13 used a context-sensitive screener (65 %), principally for those demonstrating traumatic stress reactions.

The interventions targeted post-traumatic stress symptoms (PTSS), internalizing symptoms (depression, anxiety), and behavioral and emotional problems more generally (e.g., conduct problems). Eighteen publications (78 %) reported results on multiple outcome indicators. Of the 11 publications (48 %) that have PTSS as a primary outcome, ten included multiple indicators for internalizing symptoms and behavioral and emotional problems. Two case studies examined potential mechanisms of effective counseling and were delivered on an individual basis [20, 21]. Almost all programs were group based (*n* = 18, 90 %), except for two interventions that included both group and individual elements [22••, 23••]. A multi-level multi-country program that took a public health approach was reported in eight publications [19-21, 24, 25••, 26-28]. This multi-level multi-country program targeted children with elevated psychosocial distress upon primary screening who were offered a classroom-based intervention. Those in need of more individualized or specialized care were referred for counseling and psychiatric care if available. Ten interventions involved the family or community in any capacity [21, 22••, 23••, 25••, 29-34].

### Evidence Base

All publications reported positive promotion, prevention, and treatment effects on a range of indicators. Eighteen studies (78 %) reported positive effects on their primary outcomes [16, 18, 22••, 23••, 25••, 26-35, 36••, 37, 38], and eight (44 %) of these 18 showed positive impacts on specific subgroups [16, 18, 26-28, 30, 31, 36••]. Therefore, only ten publications (43 %) reported positive overall promotion, prevention, and treatment effects on symptom reduction and improved well-being for their primary outcomes.

Improvements were shown on multiple outcome indicators for 16 (70 %) studies [15-18, 22••, 23••, 24, 25••, 26-28, 33-35, 37, 38]. Most positive effects were small or moderate in size, with a few studies reporting large effect sizes. Trauma-focused cognitive behavioral therapy (TF-CBT) was used to alleviate distress, for both sexually exploited girls and war-affected boys in Democratic Republic of the Congo, and demonstrated large effect sizes (*d* = 2.13 to 2.75 [22••], *d* = 0.64 to 2.01 [23••]). A traumatic grief psychotherapy in Palestine resulted in significant improvements in PTSS and depression symptoms also with large effect sizes (*d* = 0.62–2.38) [38].

Five (22 %) publications identified negative outcomes. Gender and abduction history interacted to moderate the effectiveness of group interpersonal psychotherapy (G-IPT) with a small negative outcome on male non-abducted subjects in regards to depression [36••]. There were increased PTSS in the experimental group compared to control, post-intervention in the student mediation program in Palestine [37]. A “Teaching Recovery Technique” aiming to improve emotion regulation (ER) and coping abilities actually established that a decrease in ER was associated with improved mental health and psychosocial well-being [17]. Gender-specific outcomes demonstrated that girls had greater reduction in PTSS in the waitlist control, compared to the experimental group in a classroom-based intervention in Sri Lanka [28]. The same intervention implemented in Burundi also reported negative effects for subgroups of children (depending on age, household composition, exposure, and displacement), with a better outcome for hope and functioning in the waitlist control compared to the experimental group [27].

### Intervention Modalities

**Fig. 2:**
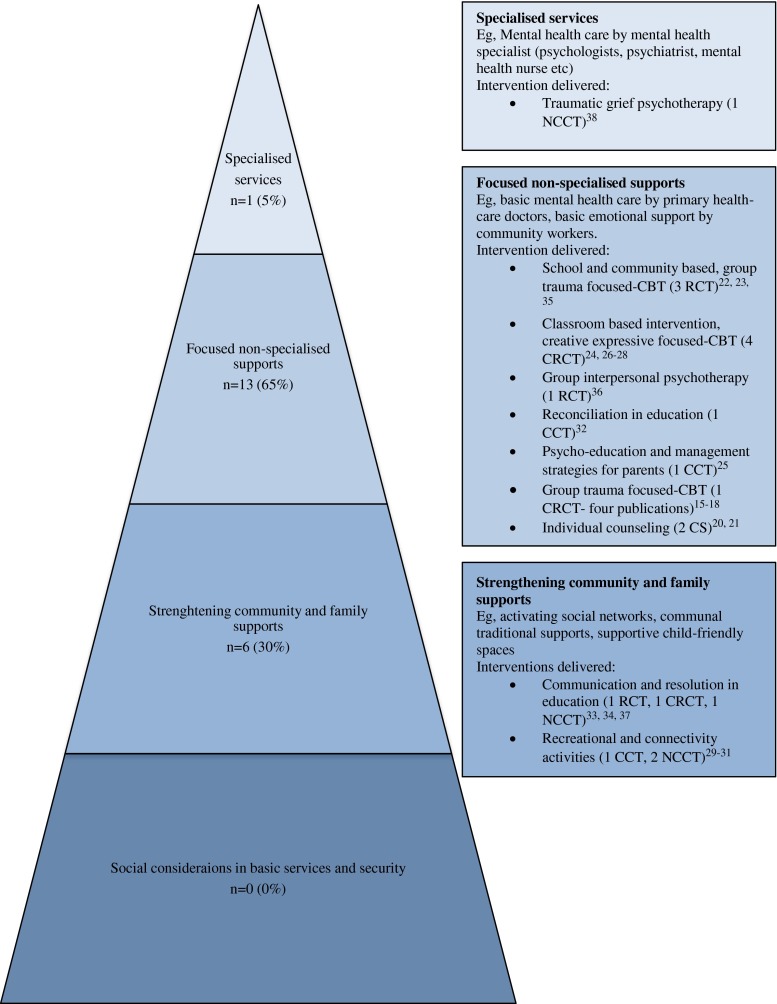
Included interventions mapped onto care framework

Figure 2 outlines the range of intervention modalities mapped on to the multi-layered approach as advocated by the IASC guidelines. The most frequently mentioned modalities were creative expressive, psycho-educational, and cognitive behavioral strategies. Creative expressive approaches emphasized interactive activities such as drama, music, role-playing, and drawing. They aim to build better relationships and improve well-being. Three interventions had a core (as opposed to inclusive) focus on creative expressive activities [29-31]. The case studies that investigated counseling mechanisms used face-to-face engagement and supportive strategies centered on empowering the participant to reduce psychological and mental health problems [20, 21]. Other publications reported psycho-education and psychotherapies as strategies to improve the mental health and psychosocial well-being of children affected by armed conflict. Psycho-educational activities were implemented in five studies that focused on resilience, stress management, and conflict resolution [25••, 32-34, 37]. The psychotherapies targeting specific psychopathology reported: trauma-focused CBT [15-18, 22••, 23••, 35], interpersonal psychotherapy [36••], traumatic grief psychotherapy [38], and combined creative expressive activities with CBT [24, 26-28].

As can be seen in Fig. 2, there are no interventions in the dataset that focused on social considerations in basic humanitarian services and security, and a large majority of the programs investigated focused non-specialized support. Most mental health and psychosocial promotion interventions were school based. Only one study focused on the top level that represents the treatment of subgroups of children diagnosed with a mental health disorder who required more focused individualized care. A grief psychotherapy in Palestine as reported above aimed to treat those who were diagnosed with PTSD [38].

There were also two case studies evaluating treatment mechanisms of counseling in Burundi and South Sudan for children in need of individual-level care; however, counseling was provided by a non-specialist (hence included in the focused care level) [20, 21]. Positive results were associated with an explicit trust formation and disclosure, active problem solving, structural trauma-focused exposure, family involvement, and parental support. Both studies reported displaying a moralistic attitude in counseling had a negative impact on a child’s mental health and psychosocial well-being.

### Within Study Bias^2^

The RCT(s) implemented in the Democratic Republic of the Congo were the only interventions to have low risk for both subsections of selection bias [22••, 23••, 33]. High risk and unclear risk of bias for blinding of the participants and personnel (performance bias) were coded in 50 % (*n* = 10) and 40 % (*n* = 8) of the interventions, respectively. Three publications (15 %) scored low for risk on blinding of outcome assessment (detection bias). Incomplete data representing loss to follow-up was coded as high risk in 45 % (*n* = 9) and unclear risk in 15 % (*n* = 3) of the interventions. Determining whether statistical results were selectively withheld from the publication was problematic to establish; consequently, reporting bias was coded as 100 % unclear risk. Other sources of bias such as contamination of controls were determined to be 60 % (*n* = 12) high risk. Of the possible 140 high, low, and unclear risks of bias scores, 40 % (*n* = 56) were unclear and 39 % (*n* = 55) of scores were considered as high risk.

### Cultural Modifications and Key Themes

With interventions delivered in nine different countries, it is important to review cultural adaptations. Instrument adaptations by translation and back translation, focus group discussions (FGD), and piloting were outlined in 60 % (*n* = 12) of the studies [20, 21, 22••, 23••, 24, 25••, 26-29, 33, 38]. Promotion, prevention, and treatment approaches were culturally modified in 40 % (*n* = 8) of the interventions [22••, 23••, 29, 32-35, 36••]. However, few gave detailed accounts of any actual adaptations made. The publications appeared to mainly report minor changes for instance translation of the manual and small alterations to session themes, making no changes to the overall structure of interventions. The interventions detailing their cultural adaptations such as culturally applicable analogies and examples throughout the modified program manual had the largest effect sizes of the review [22••, 23••]. Both were randomized controlled trials.

Although 70 % (*n* = 16) of studies reported specific intervention effects in certain subgroups, only one study tailored their intervention by splitting groups by age and gender [33]. Fifty-two percent of the publications recommended that future interventions should apply multi-levelled approaches [15, 17, 20, 22••, 23••, 24, 25••, 26-28, 33, 36••]. Eight studies used intervention approaches with a focus beyond children’s individual symptomology, incorporating community/caregiver aspects [21, 22••, 23••, 25••, 29, 30, 32, 33]. The community/caregiver aspects included helping the elderly, planting trees, psycho-educational classes for parents, a graduation ceremony attended by key figures in the community, and the creation of a community advisory board to assist with implementation. Feedback on treatment quality and satisfaction were only ascertained from children in 13 % (*n* = 3) of publications [30, 31, 35]. The delivery agent’s relationship with the participant was described as integral to positive treatment effects in both publications on mechanisms of counseling [20, 21]. However, only one study gave a rationale for their recruitment strategy [23••].

## Discussion

The results of this systematic review, covering the period 2009–2015, illustrate the current modalities and evidence of psychosocial and mental health interventions for children affected by armed conflict in LMIC.

### Summary

All 24 identified publications reported positive benefits (i.e., promotion, prevention, or treatment) associated with evaluated interventions. However, under half (43 %) of these demonstrated an overall positive impact on their primary outcomes, and five studies (22 %) displayed negative effects. Therefore, the interventions may have improved some children’s mental health but undermined the natural recovery of others. Most mental health and psychosocial interventions were school based, which as a group demonstrated mixed results. The evidence suggests that interventions often resulted in specific subgroup effects. The school-based interventions mainly reported smaller effect size impacts on symptom reduction of primary outcomes and more positive effects on secondary outcomes (mostly not disorder-specific outcomes, for example behavioral problem, or protective factors such as hope and social support). Nine different countries were included in the review; however, few cultural adaptations were made. Adapting interventions to the cultural context may lead to added impact, for example inviting community leaders and stakeholders in helping to design programs, thus being empowered with a voice in the design of the intervention that will match the communities’ needs [39]. Although numerous publications detailed moderators and mediators of MHPSS, children and adolescents were rarely consulted for their experiences. Effects of individual counseling for children were moderated by the relationship the client had with their counselor, the structure and components of care, and family involvement and support.

### Comparison

This updated systematic review detailed both differences and similarities to the previous systematic review conducted 5 years prior. There is a greater quantity of interventions evaluated in the updated review, with 24 studies identified over a 5-year period compared to a previous 12 studies (for the period 2009 and before). Using the classes of evidence by Morris that have been adapted, interventions were levels 1 or 2 for 70 % (*n* = 14) of studies, where previously it was 42 % (*n* = 5) [12]. This suggests an encouraging improvement in evidence for efficacy and effectiveness of MHPSS for children affected by armed conflict. The inclusion of stronger study designs assists in establishing causality between intervention approach and positive outcomes. However, only two of the current studies had majority low risk of bias scores; this reduces the potential validity of reported results from the included interventions [23••, 33]. Although the level of evidence from study design appears to have improved, there is still a lack of rigor for many of the evaluations. The process in which interventions are implemented also needs greater description, particularly with regards to cultural adaptation. There is a lack of clarity in reported study design, implementation, and results.

The results demonstrate moderate effect sizes in a large majority of the publications and a major focus on PTSD; this was also the case in the 2009 review. However, the current review also includes studies with large effects and most studies also assessed depression and/or behavioral problems. This may have been the result of the ongoing debate about interventions that solely focus on the individual’s conflict exposure and PTSD symptoms, without also addressing broader risk and protective systems factors [7, 9, 10]. Per illustration, in Kabul, war exposure accounted for only 15 % of the variance in PTSD symptom levels, and in Sri Lanka, it was 8 %. Similarly, direct exposure accounted for 2 % of the variance in distress levels in Palestinian youth and only 1 % of the variance in PTSD symptom levels was attributable to the violence experienced in Sudan for Darfurian refugees in Chad [10]. A daily stressors model to outline these results indicated a need to incorporate environmental and societal factors when designing interventions. Multi-levelled interventions that incorporated a community-based approach were advocated in the previous review. Although a multi-levelled care package was included in the current review, few studies implemented an intervention that aimed to integrate into the existing local health or social systems (a strategy advocated to promote sustainability [40]). Multi-level interventions were much more represented in the current review; however, these publications all stem from one multi-country program [19-21, 24, 25••, 26-28].

### Implications

There are continuing gaps in the literature that explores effectiveness of mental health and psychosocial support interventions for children affected by armed conflict. First, while most of the interventions being implemented in humanitarian settings are geared towards strengthening community support (e.g., activating social networks, supportive child-friendly spaces—level 2 in Fig. 2), most research attention still goes to focused interventions, especially CBT. More diversification in research focus is therefore called for. Few publications focus on parents and families. Given existing evidence of parenting interventions in high-income setting, and the promising results from the brief parenting psycho-education intervention in Burundi [25••], family-oriented interventions should be further explored. Specifically, as a focus on young children (below 6 years of age) is entirely lacking in the current review. Also, the role of physical activities in interventions may be further explored. Several systematic reviews have reported an association between physical activity and improved self-esteem, social interaction, and lower levels of depression and anxiety for children [41-43].

Second, although we find that there are overall positive benefits of evaluated intervention for subgroups of children, we also identified studies with negative impacts. In our opinion, this calls for better targeting and improved conceptual development of interventions. The described interventions often consisted of multiple elements and often focused on children in settings of armed conflict broadly. Nevertheless, publications rarely outlined how specific intervention elements were aimed at addressing specific paths of a causal pathway. Few intervention descriptions described a theory of change that outlined how interventions may affect different subgroups of children, even though interventions were implemented in complex and dynamic environments with ongoing adversity threatening mental health and well-being. Similarly, we found that interventions that had very different goals (ranging from promotion, to prevention, to treatment) often applied the same outcome measures, rather than select outcome measures that matched intervention goals. We feel that further sophistication in tailoring interventions to specific socio-cultural contexts and conflict settings could help mitigate the negative effects that were identified in the identified studies.

### Limitations

A few limitations of the systematic review methodology should be noted. The review searched three databases and only peer-reviewed articles were included, excluding any gray literature. Primary outcomes were often not stated therefore they were assigned to categories based on the core intervention focus. Furthermore, publications targeting children and adults simultaneously were excluded if the majority of the sample was adult at the time of intervention, yet they still may yield important findings for the review. For example, a community-implemented intervention using narrative exposure therapy demonstrated positive results on former child soldiers even after a 1-year follow-up period [44]. The Cohen’s *d* effect sizes are established from a single post-intervention time point, neglecting natural recovery and baseline differences between groups. Cohen’s *d* effect sizes can therefore be an under- or overestimate of the intervention effect.

## Conclusion

Although the number of publications and level of evidence have improved, there is still a general lack of rigor and clarity in study and intervention design and reported results. Some interventions show promising results demonstrating mostly moderate effect sizes on mental health and psychosocial well-being, albeit often for subgroups. CBT-based interventions, and the school as the delivery platform, are the most commonly reported. There is a need for increased diversification in research focus, with more attention to interventions that focus at strengthening community and family support, and to young children, and improvements in targeting and conceptualizing of interventions.

## Appendix 1

Table 2

**Table 2:** Inclusion and exclusion criteria for review

	Included	Excluded
Publication type	Date (1 January 2009) to (20 July 2015) Only lower and middle-income countries (LAMIC) Only studies reported in English peer-reviewed journals	
Study design	All study designs	
Study population	Child and adolescent population affected by armed conflict	Adult refugees in high-income countries (HIC)
Definition mental health	State of well-being in which every individual can cope with the stresses of life, can work productively, and is able to make a contribution to their community [45]	
Definition of mental health and psychosocial support (MHPSS)	MHPSS is used to describe any type of local or outside support that aims to protect or promote psychosocial well-being and/or prevent or treat mental disorder [4]	Interventions not evaluated MHPSS not specific to study population
Definition of child and adolescent	A person of age 18 or below	
Definition of armed conflict	Whenever there is a resort to armed force between states or protracted armed violence between governmental authorities and organized armed groups or between such groups within a state [46]	Non-war-related violence Natural disasters
Outcome	Clinical outcomes Psychosocial outcomes	

## Footnotes

1 When calculating percentages of *interventions*, we have used *n* = 20 as four publications report on the same intervention and one case study reported multiple interventions. For the calculation of percentages of *publications*, *n* = 23 is used, not including the one with multiple results on multiple interventions.

2 Four studies used one data set; study bias was recorded from the original publication [18]; therefore, percentages are based on *n* = 20 (publication with multiple interventions excluded)*.*
